# Novel Germline c.105_107dupGCT *MEN1* Mutation in a Family with Newly Diagnosed Multiple Endocrine Neoplasia Type 1

**DOI:** 10.3390/genes11090986

**Published:** 2020-08-24

**Authors:** Magdalena Stasiak, Marek Dedecjus, Katarzyna Zawadzka-Starczewska, Emilia Adamska, Monika Tomaszewska, Andrzej Lewiński

**Affiliations:** 1 Department of Endocrinology and Metabolic Diseases, Polish Mother‘s Memorial Hospital-Research Institute, 93-338 Lodz, Poland; mstasiak33@gmail.com (M.S.); kasiula891@op.pl (K.Z.-S.); emila0079@gmail.com (E.A.); 2 Department of Endocrine Oncology and Nuclear Medicine, Maria Sklodowska-Curie National Research Institute of Oncology (MSCNRIO), 02-781 Warsaw, Poland; marek.dedecjus@gmail.com; 3 Department of Pediatrics, Oncology, Hematology and Diabetology, Central Teaching Hospital of the Medical University of Lodz, 91-738 Lodz, Poland; nickus@me.com; 4 Department of Endocrinology and Metabolic Diseases, Medical University of Lodz, 93-338 Lodz, Poland

**Keywords:** multiple endocrine neoplasia type 1, *MEN1* gene, menin, primary hyperparathyroidism, pituitary adenoma

## Abstract

In multiple endocrine neoplasia type 1 (MEN1), the causative *MEN1* gene mutations lead to the reduced expression of menin, which is a tumor suppressor protein. In this study, we present a case of a 16-year-old woman with severe primary hyperparathyroidism and a non-functioning pituitary microadenoma. Genetic testing demonstrated a novel germline heterozygote variant c.105_107dupGCT of *MEN1*, leading to Leu duplication in position 37 of the menin polypeptide chain. As such a mutation was not reported before as a causative one, confirmation of its pathogenicity required showing the same mutation in a symptomatic first-degree relative. An identical mutation was found in the patient’s father, who was further diagnosed with hyperparathyroidism and a pituitary microadenoma. We observed the presence of the same MEN1-related tumors but an entirely different symptom severity. To the best of our knowledge, this is the first report of MEN1 syndrome caused by the c.105_107dupGCT *MEN1* mutation. This case report demonstrates the importance of genetic evaluation towards MEN1. Genetic testing for MEN1 mutations should be performed in all patients with MEN1-related tumors, and in the young patients even with only one such tumor, despite the supposedly negative family history.

## 1. Introduction

Multiple endocrine neoplasia type 1 (MEN1) is an autosomal, dominantly inherited syndrome predisposing to the development of many different endocrine and non-endocrine tumors, mainly parathyroid adenomas, gastroenteropancreatic neuroendocrine tumors (GEP-NETs), and the anterior pituitary. The clinical diagnostic criteria include the presence of at least two of those three main tumor types [1]. The younger the patient is at the symptom onset, the more strongly MEN1 syndrome should be suspected. The incidence ranges from 1:10,000 to 1:100,000 [1]. A geographic clustering, as a result of a founder’s effect, has been described [1]. The penetrance of the MEN1 syndrome is high. In about 50% of patients, symptoms occur by 20 years of age, more than 95% by 40 years of age, and virtually 100% by 60 years of age [2,3]. In a patient with one affected first-degree relative, the presence of only one MEN1-related main tumor is sufficient for the clinical diagnosis [1,4]. 

The *MEN1* gene is a tumor suppressor gene located on chromosome 11q13. It contains 10 exons and encodes a 610 amino-acid protein—menin [4]. Menin is a nuclear protein involved in many molecular interactions related to transcriptional regulation, genome stability, proliferation, and cell division [1,5]. For the endocrine tumorigenesis, the most important significance of menin is the regulation of *CDKN1B* and *CDKN2C* encoding cell cycle proteins p27 and p18 [6,7]. The first exon of the *MEN1* gene and a part of exon 10 are not translated. The main transcript of 2.8 kb was found in many human tissues, and an additional 4 kb transcript has been described in the pancreas and thymus, which suggests tissue-specific alternative splicing [8]. More than 90% of tumors in MEN1 patients result from a loss of heterozygosity (LOH) [5]. Over 1300 *MEN1* mutations have been reported, scattered in and around the open reading frame without significant grouping, mainly in coding exons but also in intron sequences, and without any significant hot spots [5,9]. Most *MEN1* germline mutations (69%) are predicted to be pathogenic due to the premature menin truncation caused by the frame-shift mutations (42%) and nonsense mutations (14%), or exon region deletions, which are attributed to splicing defects (10.5%) and large deletions (2.5%) [10]. Other *MEN1* germline mutations include missense mutations (25.5%) and a single or a few amino acid in-frame deletions or insertions (5.5%) [10]. More than two-thirds of all the identified mutations (frameshifts, nonsense, and some splicing site mutations) cause a loss of menin function. The remaining 20–30% of *MEN1* mutations are missense mutations and in-frame deletions, potentially affecting the interaction-site of menin partners, changing the ability of menin to regulate target promoters, or promoting rapid proteolytic degradation of menin. Missense menin transcripts are present at reduced levels, which suggests a role of rapid proteolytic cleavage [11].

Genetic testing should be performed in all patients with MEN1-related tumors. The confirmation of a *MEN1* mutation should be followed by the family screening. In the case of a mutation not reported before to be a causative one—especially other than the frame-shift mutation, nonsense mutation, or large deletions—confirmation of its pathogenicity requires showing the same mutation in another affected first-degree relative. 

Biochemical tests are required in all asymptomatic, genetically affected relatives. Biochemical testing may reveal MEN1-related tumors even 10 years before the disease becomes clinically evident, allowing for early surgical intervention [4] and prevention of permanent complications. 

In this case report, we present a novel heterozygote c.105_107dupGCT *MEN1* mutation, leading to leucine (Leu) duplication in position 37 of the polypeptide chain of menin. 

## 2. Case Presentation

### 2.1. Patient’s Description 

A 16-year-old female was referred to our department in March 2018 for further investigations regarding the management of her previously identified primary hyperparathyroidism (PHP), which was revealed in the course of diagnostics of the recurrent nephrolithiasis. The patient had suffered from several episodes of renal colic since 2016, and finally, in December 2017, she underwent a shock wave lithotripsy curative procedure. Additionally, in October 2017, osteopenia was revealed, with lumbar bone mass density (BMD) 0.813 g/cm^2^ and Z-score of −1.5. At the time of PHP diagnosis, serum calcium concentration was 2.95 mmol/L (11.8 mg/dL), parathyroid hormone (PTH) 172.2 ng/L and phosphorus 0.646 mmol/L (2.0 mg/dl). The most important laboratory results of the patient’s tests are presented in Table 1. A ^99^Tc MIBI SPECT/CT was performed in December 2017 but it did not reveal any pathologic focal uptake. Treatment with 30 mg of cinacalcet daily was introduced, with only a slight reduction in serum calcium and PTH level (Table 1). Thus, the dose was increased to 45 mg daily, but the normalization of calcaemia was not achieved (Table 1). Serum creatinine levels were always within the normal range since the time of diagnosis. 

**Figure 1:**
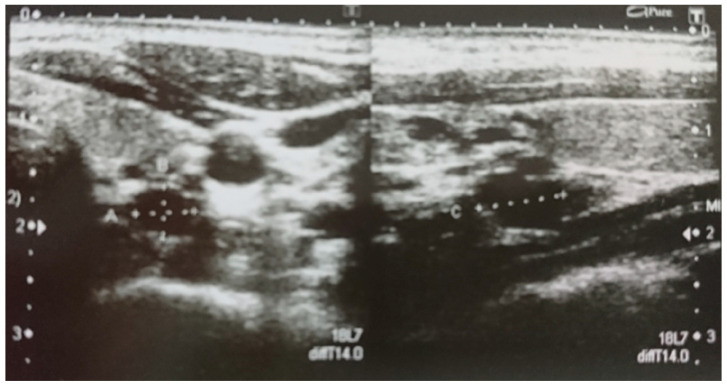
Sonographic image of left-inferior parathyroid adenoma before the 1st surgery.

**Figure 2:**
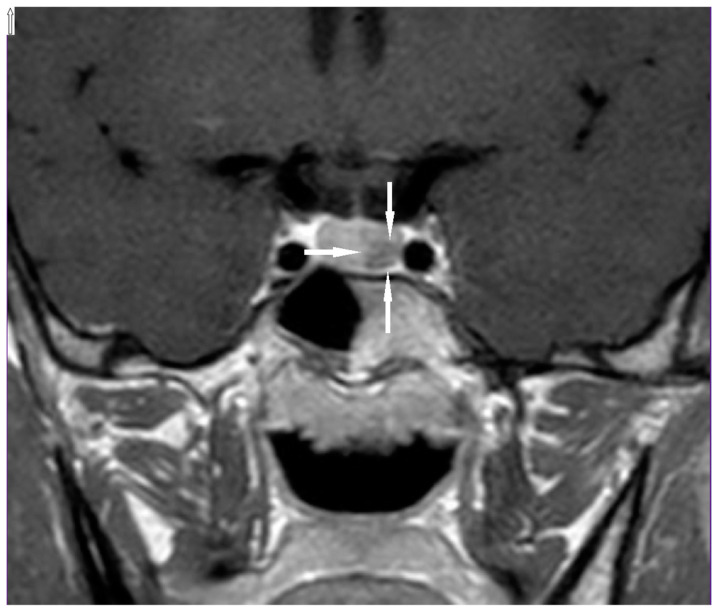
Pituitary microadenoma (arrows) visible in the MRI of the proband.

On admission to our department, a physical examination did not reveal any abnormalities. In a thyroid ultrasound examination (US), a hypoechogenic structure 5x4x8 mm was detected below the left thyroid lobe (Figure 1). An ultrasound-guided fine-needle aspiration biopsy (FNAB) was performed and confirmed the presence of a left inferior parathyroid adenoma, both cytologically and by measurement of PTH in the FNAB needle washout. Due to the young age of the patient, screening for endocrine tumors was performed and a pituitary magnetic resonance imaging (MRI) showed a lesion corresponding to a microadenoma (Figure 2). Serum prolactin and other pituitary hormonal parameters were within the normal ranges. Non-functioning pituitary adenoma was diagnosed. In accordance with the guidelines and literature data, the patient was referred to *MEN1* germline mutation testing. A never-before-reported heterozygous germline mutation c.105_107dupGCT was detected, so all first-degree relatives were tested to confirm the pathogenicity of this variant. 

The same mutation was detected in the patient’s father, who had never been diagnosed with any of the MEN1 components. Thus, the patient’s father was admitted to our department, and a medical history of all family members was taken. This patient was asymptomatic, with a negative history of gastrointestinal diseases, nephrolithiasis, osteoporosis, and endocrine disorders. However, serum biochemisty revealed hypercalcaemia and an elevated PTH level (Table 1). A neck US did not allow us to localize parathyroid adenoma/hypertrophy. MRI scans showed a pituitary microadenoma. Pituitary hormonal parameters were within the normal range. The US of the abdomen did not reveal nephrolithiasis, and the abdominal MRI did not show any lesions in the pancreas. 

The patient and her father were diagnosed with MEN1, and the pathogenicity of the new mutation was confirmed. 

As the genetic testing of the young woman and her family members was time-consuming and her hypercalcaemia responded poorly to cinacalcet therapy, in July 2018, microinvasive surgical excision of the localized parathyroid adenoma was performed. Histopathological examination confirmed a benign adenoma of the left parathyroid gland, sized 14 × 7 × 6 mm. After surgery, serum calcium and PTH level were only slightly reduced (Table 1), and within a few days they returned to preoperative values. The dose of cinacalcet was increased to 90 mg per day. 

In June 2019, she was readmitted to our department. A neck US revealed a lesion 5 × 4 × 13 mm, and an FNAB confirmed another left-inferior parathyroid gland. The MRI of the pituitary did not show any progression of the microadenoma. No solid lesion in the pancreas was detected using an abdominal MRI. In October 2019, the recurrence of nephrolithiasis was detected sonographically. As MEN1 was genetically confirmed at that time, the patient was referred for neck surgery. Intraoperatively, three hypertrophic parathyroid glands were found—two of them, and two-thirds of the third one, were excised. Parathyroid fragments were frozen and preserved to protect the patient from possible hypoparathyroidism. Since the second surgery, serum PTH and calcium levels have been normal (Table 1). 

### 2.2. Material and Methods

DNA was extracted from the peripheral blood samples of the proband and her first-degree relatives. 

A new generation sequencing method was applied, using an Illumina TruSight One Sequencing Panel, Ilumina NextSeq 550 sequencing instrument, according to the protocol 2 × 150 bp. The analysis was performed using the Variant Studio 3.0, IGV 2.3 software. The obtained result was confirmed by Sanger’s method.

### 2.3. Consent Procedures

The patient, her father, and all diagnosed family gave their informed written consent for all the procedures performed. Additionally, as the patient was 16 years old at the moment of her first admission, the patient’s mother also gave her written consent for all the procedures. All of them signed a consent to the publication of their medical data. The consent form was accepted by the Institute Bioethics Committee (approval code 54/2019).

## 3. Discussion

The clinical diagnosis of the newly recognized MEN1 syndrome is based on the identification of neoplastic disease in at least two of the typically affected organs, e.g., the parathyroid gland, anterior pituitary, and/or the pancreas. In our young female patient, the first manifestation of MEN1 was primary hyperparathyroidism, while a non-functioning pituitary adenoma was found during further evaluation. Genetic testing revealed a novel heterozygous germline c.105_107dupGCT *MEN1* mutation. An identical mutation was found in the patient’s father, in whom no clinical symptoms of MEN1 had been found before. *MEN1* germline mutations—including frame-shift mutations, nonsense mutations, exon region deletions, or large deletions—are predicted to be pathogenic due to the premature menin truncation. To definitely confirm the pathogenicity of other mutations, especially the mutation that had never been described as a causative one before, the confirmation of MEN1 clinical manifestation in other carriers was required. In the patient’s father, we found the same clinical features of MEN1 as in his daughter. Thus, we could confirm the pathogenicity of the new mutation. However, the severity of symptoms was completely different, despite the same genetic background. His disease was subclinical at the age of 47, while she had severe complications of hypercalcemia at the age of 16. The similarity is that, in both of them, MEN1 manifested as the PHP and non-functioning pituitary microadenoma.

Significant phenotypic variation in onset age, clinical manifestations, severity of disease, and tumor types has been described before, even in the same family having the same *MEN1* gene mutation. Many attempts to compare the clinical features in patients and their families carrying the same mutations confirmed the lack of direct phenotype–genotype correlations [5,12,13,14].

However, some authors provided arguments on the possible existence of phenotype–genotype correlations in particular cases. Soczomski et al. [15] showed a statistically significant 3.5-fold higher risk of a pituitary adenoma in patients with a frameshift mutation with the STOP codon of the *MEN1* gene. In this study, primary hyperparathyroidism, gastroenteropancreatic neuroendocrine tumor, and pituitary adenoma developed in 90, 52, and 47% of patients, respectively [15]. Longuini et al. [16] reported that a specific variant of the *CDKN1B* gene (the gene whose inactivating mutation leads to MEN4 syndrome) can modify a disease course in MEN1 patients with truncating *MEN1* mutations, resulting in a higher number of MEN1-related tumors [16]. In the study by Kovuaraki et al. [12] all patients with *MEN1* frameshift mutations had GEP-NETs. Another study revealed a higher rate of malignant tumors in patients with mutations in *MEN1* exons 2, 9, and 10 [17]. Similarly, Palermo et al. [18] discovered a new germline truncating mutation of the *MEN1* gene at exon 10, in an individual with an aggressive clinical course of GEP-NETs. A study by Thevenon et al. [19] reported a two-fold higher risk of death in individuals with a heterozygous *MEN1* pathogenic variant that affects the JunD interacting domain of menin, and it is known that the main reasons for death in MEN1 patients are malignant NETs. 

Moreover, de Paoli-Iseppi et al. [20] investigated clinical consequences of the *MEN1* gene promoter methylation, with minimal methylation observed in all patients at CpG sites 1–23. In contrast, hypermethylation was observed at CpG sites 24–31 in patients with MEN1, which was not seen in patients with parathyroid disease other than MEN 1. The average methylation at 24–31 sites was significantly correlated with age of the first parathyroid surgery [20].

Additionally, the epigenetic mechanisms triggered by environmental factors were suggested to influence the phenotype in patients with the same *MEN1* mutation [21]. 

On the other hand, no genotype–phenotype correlation has been consistently confirmed in other studies [22]. Evaluation of unrelated kindreds exhibiting the same *MEN1* mutation showed large variability of different associated tumors [2,23]. Additionally, there are reports of identical twins with identical *MEN1* mutations but with different MEN1 clinical phenotypes [24,25,26]. Finally, particular *MEN1* mutations can be associated with isolated hyperparathyroidism in some families, while other families with the same mutations develop a full MEN1 spectrum [5].

Our patients had the same clinical manifestation but different severity of symptoms and probably a different onset age. The exact assessment of the onset age in the patient’s father is impossible as he had no previous measurement of serum calcium or PTH ever performed. Similarly, no previous head/pituitary imaging was available. Neither in the patient, nor in her father, was any pancreatic tumor detected. Taking into account the presence of the two most common MEN1-related tumors in our kindred, it is not possible to speculate on the potential correlation between the novel c.105_107dupGCT *MEN1* mutation and a specific MEN1 phenotype. As was discussed above [5,21,24,25,26], the differences in the severity of symptoms and in the course of the disease are frequently observed even in the families with the same *MEN1* mutation. Some other factors, including environmental and epigenetic ones [21], might influence this phenomenon. One could suspect some influence of the gender in our cases, but no such association has been described so far. Careful surveillance is required to reveal whether our patient or her father will develop other MEN1-related tumors.

Despite the progress in the diagnosis and treatment of MEN1-associated tumors, patients still have decreased life expectancy, primarily due to malignant NETs [27]. The last clinical practice guidelines for MEN1 [28] underlined the need for early genetic and clinical diagnosis of MEN1. In our patient, the diagnostic procedures towards MEN1 was started, although she had only one component of MEN1 syndrome. We believe that in all patients younger than 40 years old, the presence of even one MEN-related tumor should constitute an indication for further diagnostic procedures. Particularly in young patients with primary hyperparathyroidism, the genetic testing should be considered even in the absence of other MEN1-related tumors. The experts recommend an intensive surveillance for both symptomatic patients with MEN1 and asymptomatic carriers, starting at the age of 5 years [27,28]. Periodical screening and clinical follow-up are recommended in all MEN1 patients, in order to introduce appropriate and early medical/surgical interventions [27]. The aim of such an approach is to ultimately decrease disease-specific morbidity and mortality. Mutation-dependent surveillance is not possible currently, because—as it was discussed above—no clear genotype–phenotype correlation can be unequivocally confirmed. The knowledge about all detected causative mutation facilitates the diagnosis and speeds up the proper treatment. Thus, the confirmation of every new causative mutation is extremely important, particularly if the mutation—like this one—is not typically related to premature menin truncation. 

## 4. Conclusions

To our knowledge, this is the first report of MEN1 syndrome caused by the c.105_107dupGCT *MEN1* mutation. The proband and her father had the same mutation, and the same MEN1-related tumors, but different severity of symptoms. This finding suggests that the identification of mutations might be very important for a prompt diagnosis and for a monitoring of family members carrying the same mutation. Genetic testing for *MEN1* mutation should be performed in all patients with MEN1-related tumors, and in the young patients even with only one such tumor, despite the supposedly negative family history. Genetic testing should always be offered to the family members of the affected patient to identify further gene carriers. Mutation-dependent surveillance is not possible, as the disease course may be different even in patients with the same mutation. 

## Figures and Tables

**Table 1:** Results of the patient’s laboratory tests from the time of diagnosis until the most recent assessment, and the lab results of the patient’s father at the time of diagnosis.

Parameter (Reference Range) [unit]	At the Time of Diagnosis	30 mg of Cinacalcet	Before the 1st Surgery 45 mg of Cinacalcet	After the 1st Surgery (June 2018)	Before the 2nd Surgery (June 2019) 90 mg of Cinacalcet	After the 2nd Surgery (January 2020)	The Patient’s Father (July 2019)
PTH (15–65) [ng/L]	172.2	136.4	84.59	78.3	88.9	21	112.6
Ca (2.20–2.65) [mmol/L]	2.95	2.85	2.73	2.8	2.62	2.20	2.65
P (0.97–1.81) [mmol/L]	0.646	0.872	1.29	NA	1.22	NA	0.90

Abbreviations: Ca, calcium; NA, not available; P, phosphorus; PTH, parathyroid hormone.
